# Association Between Candida albicans and COVID-19 in Complete Denture Wearers: An Observational Study

**DOI:** 10.7759/cureus.47777

**Published:** 2023-10-27

**Authors:** Mohd Osman Ali, Babashankar Alva, Suresh Nagaral, Rohit Patil, Mohammad Ullah Khan, Durgesh A Tiwari

**Affiliations:** 1 Department of Dentistry, Deccan College of Medical Sciences, Hyderabad, IND; 2 Department of Dentistry, Faculty of Dentistry, Ramaiah University of Applied Sciences, Bangaluru, IND; 3 Department of Prosthodontics, JMF's ACPM Dental College, Dhule, IND; 4 Department of Dentistry, Tobal Dental Clinic, Buraydah, SAU; 5 Department of Conservative Dentistry and Endodontics, Yogita Dental College and Hospital, Khed, IND

**Keywords:** corelation, colony forming unit, complete denture, covid 19, oral candidiasis

## Abstract

Introduction

The phenomenon of coronavirus disease 2019 (COVID-19)-related candidiasis is gaining increased attention and acknowledgment as an integral component of the severe consequences of COVID-19. The aim of the present study was to assess the association between *Candida albicans* and COVID-19 in complete denture wearers.

Materials and methods

An observational study was conducted on 45 complete denture wearers, who were divided into three groups as follows: Group 1, 15 subjects with mild to moderate COVID-19 infection; Group 2, 15 subjects with severe COVID-19 infection; and Group 3, 15 subjects without COVID-19 infection. Mean colony forming units (CFU) were observed on agar plates containing Sabouraud dextrose in the salivary samples of the participants. Analysis of variance, followed by post-hoc analysis by Tukey’s test, was used to compare CFU between the groups. Pearson’s correlation coefficient was used to study the correlation between variables.

Results

The highest average colony-forming units of Candida albicans were observed in Group 2, followed by Group 1, compared to the control group, and a significant (p<0.001) difference was found. A weak positive correlation was found between the age of the patients and the duration of denture usage, as well as between age and the counts of *Candida albicans* in Groups 1 and 3. This correlation was more pronounced in Group 3. A strong positive correlation was observed in all groups between the *Candida albicans* count and the duration of denture usage by the patients.

Conclusion

The association between *Candida albicans* and denture wear was compounded by the presence of COVID-19. Consequently, the timely identification of *Candida albicans* infection in patients with COVID-19 is important to establish more efficacious approaches for antifungal treatment and prophylactic interventions.

## Introduction

The viral illness known as Coronavirus disease 2019 (COVID-19) is characterized by its high level of contagion and is caused by the severe acute respiratory syndrome coronavirus 2 (SARS-CoV-2). The global impact of COVID-19 is devastating, with a staggering number of over 6 million fatalities reported worldwide [1]. Previous investigations have indicated that SARS-CoV-2, which is the coronavirus responsible for the condition known as COVID-19, possesses the capability to actively invade cells that compose the lining of the oral cavity and salivary glands. The newfound discoveries have the potential to elucidate why COVID-19 can be detected through the analysis of saliva, and approximately half of all cases of COVID-19 exhibit symptoms pertaining to the oral region, including the loss of gustatory perception, xerostomia, and the development of oral ulcers. These outcomes also suggest that the oral cavity and the secretion of saliva may serve as pivotal and often overlooked mechanisms for the diffusion of SARS-CoV-2 throughout the entirety of the human body, and conceivably, for its transmission from one individual to another [2,3]. Oral microorganisms are not only linked to the emergence of oral ailments, such as denture stomatitis caused by Candida, periodontitis, or caries, but they can also contribute to an increased susceptibility to numerous systemic afflictions, including aspiration pneumonia, gastrointestinal infection, pleural infection, and bacterial endocarditis. Moreover, infectious procedures in the oral cavity can instigate a systemic inflammatory process and hence influence various organs and pathologies [4].

Dental prostheses possess the capacity to serve as a reservoir for microorganisms, and the occurrence of elderly individuals who utilize dental prostheses is typically substantial [5]. Candida infection is very common in maxillary denture wearers, which might be due to many factors, such as reduced salivary flow, constant friction between the palate and denture, and poor oral hygiene, and it is further aggravated by the presence of systemic conditions such as diabetes and hypertension [6]. Furthermore, fungal organisms belonging to the *Candida* genus are widely regarded as opportunistic microorganisms, and patients who are confined to hospitals exhibit a heightened susceptibility to oral candidiasis due to alterations in both the environment and systemic conditions, resulting in disruptions to the natural microbiota and facilitating the onset of opportunistic infections [7].

COVID-19 patients are likely to experience multiple therapeutic interventions, which have the potential to further enhance their vulnerability to infection. These treatments encompass various medical procedures, such as admission to the intensive care unit (ICU), administration of broad-spectrum antibiotics and corticosteroids, and the requirement for intubation. Furthermore, pre-existing chronic conditions, including diabetes and hypertension, exacerbate the immunocompromised state of these patients. Collectively, these factors significantly increase the susceptibility of SARS-CoV-2 patients to the onset of oral and oropharyngeal candidiasis [8].

To date, no research has been conducted to evaluate the association between candida albicans and COVID-19 in individuals wearing complete dentures. The null hypothesis of the current study posits that there is no disparity in the presence of *Candida albicans* between denture wearers who have contracted COVID-19 compared to those who have not. Hence, the objective of the present study was to examine the correlation between COVID-19 and *Candida albicans* load in individuals who wear complete dentures.

## Materials and methods

The study included two components: a case-control and a prospective design. The research was carried out through a collaborative effort between the Jawaharlal Memorial Foundation (JMF) Medical College and Hospital, Dhule, Maharashtra, and the Department of Prosthodontics, JMF Dental College, Dhule. The case-control part of the study spanned from March 2021 to December 2021, whereas the prospective part of the study spanned from November 2022 to January 2023. The study was approved by the institutional ethical committee (EC/NEW/INST/2022/2959/2022/076), and adhered to the principles outlined in the Declaration of Helsinki. Furthermore, the study adhered to the Strengthening the Reporting of Observational Studies (STROBE) guidelines [9]. Written informed consent was obtained from all participants prior to the commencement of the study. Furthermore, any form of data revealing the identities of the subjects was kept confidential.

Sample size calculation

The sample size was calculated using G*Power software (version 3.1, University of Dusseldorf, Germany). The formula used for analysis was (Z-score)^2^ × standard deviation (SD) x (1-SD) / (margin of error)^2^. The power of the study was 90% and the margin of error was 5%. The standard deviation for the unknown population was 50%. The expected sample size was 45.

Study design and eligibility criteria

The study was planned for 45 complete denture wearers who were explained in detail about the study and gave their informed consent to participate in the study. Thirty patients were diagnosed with COVID-19 positive based on real-time reverse transcription-polymerase chain reaction (RT-PCR) and laboratory reports, including plasma levels of white blood cells (WBCs), platelets, C-reactive protein (CRP), aspartate aminotransferase (AST), alanine aminotransferase (ALT), γ-glutamyl transpeptidase (GGT), alkaline phosphatase, and lactate dehydrogenase (LDH). The data for these patients were obtained from a medical hospital between March 2021 and December 2021. The control group consisted of 15 patients who tested negative for COVID-19. A total of 280 records were screened to select study groups based on their eligibility criteria.

Inclusion and exclusion criteria

Subjects aged 50-60 years, both males and females, and complete denture wearers of at least the maxillary arch for more than five years, who gave their consent to participate, who had not taken any systemic antibiotics or antifungal drugs in the past six months, and free from oral candidiasis symptoms were included in the study. The patients who had systemic conditions such as diabetes, hypertension, or cardiovascular disease, subjects who had xerostomia, pregnant or lactating females, subjects who were on immunosuppressants, subjects who had taken anti-fungal drugs, steroids, anti-viral drugs, and smoking in past six months, were excluded from the study. The inclusion criteria for the test group were as follows: a positive history of COVID-19 between March 2021 and December 2021.

Detailed data were obtained, including demographics, COVID vaccination schedule, medical records comprising laboratory tests, RT-PCR reports, clinical findings, radiological findings based on computed tomography scans or chest X-rays, period of hospitalization, and course of treatment offered. Apart from this, their dental history was also recorded in detail, including the time of denture delivery, type of denture delivered, method used to clean the denture, hours of wear, any past oral infections such as denture stomatitis, last dental visit, oral hygiene methods, and type of diet. All participants were asked about the presence of symptoms of COVID-19 at present or in the past month, such as fever, cough, shortness of breath, headache, and fatigue. None of the patients reported any symptoms of COVID-19 at present.

Group allocation

Thirty patients with positive COVID-19 history were divided into two groups: Group 1 - 15 patients with mild to moderate COVID-19, and Group 2 - 15 patients with severe COVID-19, who were hospitalized but did not require mechanical ventilation based on their medical records provided. These patients were compared with the control group: Group 3 - 15 patients without COVID-19 infection history.

COVID-19 treatment protocol which was followed in the test group

The treatment protocol of the Government of India (Ministry of Health and Family Welfare) was followed for the treatment of COVID-19 patients. Patients with mild to moderate COVID-19 patients had uncomplicated respiratory tract infection, with symptoms such as fever, cough, sore throat, nasal congestion, headache, weakness, saturation of peripheral oxygen (SpO2) levels >90%, and no signs of pneumonia on chest X-ray or computed tomography (CT) scans. They were treated at home with symptomatic treatment for fever using antipyretics, maintenance of hydration, steam inhalation, warm saline rinses, and regular monitoring of temperature and saturation of peripheral oxygen (SpO2) levels. Severe COVID-19 patients had clinical symptoms of COVID-19, signs of pneumonia on chest X-ray or CT scan, respiratory rate >30 breaths/min, and respiratory distress (SpO2<90%). They were treated with antiviral drugs, methylprednisolone injection, supplemental oxygen support, intravenous fluid management, and injection of low-molecular-weight heparin, in addition to symptomatic treatment.

Collection of salivary samples for detection of *Candida albicans*

**Figure 1:**
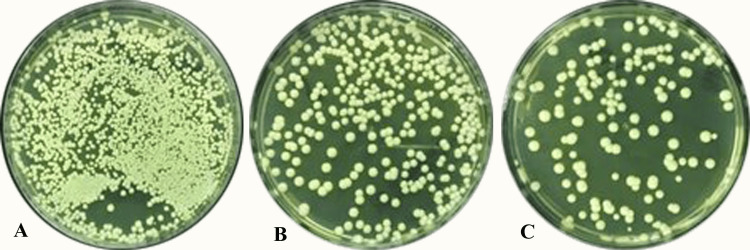
Candida albicans colony in Sabouraud dextrose agar media at 10X magnification A. In patients with a severe COVID-19 infection history; B. In patients with a mild COVID-19 infection history; C. In patients without a COVID-19 infection history

The participants were given explicit instructions to abstain from consuming food or beverages for a duration of two hours prior to the commencement of the sample collection procedure. Unstimulated saliva (no less than 2 ml) was collected in sterile tubes during the clinical sitting. All tubes were coded for identification. Subsequently, the collected saliva was expeditiously preserved at a temperature of 4 ^o^C by means of dry ice packs and subsequently conveyed for the purpose of microbiological analysis. *Candida albicans* was subjected to aerobic cultivation at a temperature of 30 ºC on agar plates containing Sabouraud dextrose (SDA, Oxoid, Hampshire, UK) medium as shown in Figure 1. Following a two-day period, the colonies were counted in a digital colony counter at 10X magnification.

Blinding

This was a double-blind study, in which the investigator who collected the salivary samples and the microbiologist were blinded to the group allocation.

Reliability

The colony-forming units (CFU) were individually enumerated by two experienced microbiologists. The mean value was calculated based on the combined scores. The interobserver reliability was determined to be 0.92, as measured by the kappa coefficient. The intra-observer reliability was assessed by asking the observers to score randomly selected 15 samples after one week. They were blinded to the initial scores. The intra-observer reliability was found to be 0.88. 

Statistical analysis

Data are presented as means and standard errors and were statistically analyzed using SPSS Statistics (version 22.0; IBM Corp., Armonk, USA). The normal distribution of the data was studied using the Shapiro-Wilk test. As data were found to be normally distributed, Analysis of Variance (ANOVA) was used to study differences between the groups, followed by post-hoc analysis using Tukey’s test. Pearson’s correlation coefficient was used to study the correlation between the variables. All levels of significance were set at p < 0.05.

## Results

There were no significant differences in the baseline characteristics of the study groups, such as age and mean duration of denture use, which negates the confounding bias due to differences in age and number of months of denture use (p>0.05). There was an equal distribution of males and females in all groups (eight males/seven females). The mean age of the participants was 55 years, and the mean duration of the denture was 3.5 years, as shown in Table 1.

**Table 1:** Demographic characteristics of the study groups NS: Not significant; SD: Standard deviation

Variables	Group 1 (Mean±SD)	Group 2 (Mean±SD)	Group 3 (Mean±SD)	p-value
Age (years)	55.73±2.01	55.21±2.85	57.33±2.58	0.068 (NS)
Duration of denture use (months)	34.33±9.61	33.82±10.53	39.80±7.17	0.221 (NS)

The null hypothesis was rejected in our study, as statistically significant differences were observed between the groups for the mean colony-forming units (CFU) of *Candida albicans* (p<0.001). The patients who suffered from severe COVID-19 had the maximum count of *Candida albicans *(107.9±7.95 CFU), followed by patients who had mild to moderate COVID-19 (76.86±8.65 CFU), compared to the control group (52.13±9.21 CFU), as shown in Tables 2, 3.

**Table 2:** Comparative analysis of mean colony-forming units between different groups using Analysis of variance (ANOVA) *p<0.0001: Highly significant; SD: Standard deviation

Groups	Colony forming units (CFU) (Mean±SD)	p value
Group 1	76.86±8.65	0.0001*
Group 2	107.9±7.95	
Group 3	52.13±9.21	

**Table 3:** Intra-group comparison of mean colony-forming units using post hoc Tukey’s test *p<0.001: Moderately significant

Groups	Mean difference	Standard error	p value
Group 3 vs Group 1	24.733	11.113	0.001*
Group 3 vs Group 2	55.767	25.057	0.001*
Group 1 vs Group 2	31.033	13.944	0.001*

In both Groups 1 and 3, a weak positive correlation was observed between age and duration of denture usage. Conversely, Group 2 exhibited a moderately positive correlation, indicating that with advancing age, there was a corresponding increase in the length of time that dentures were used. A slight positive correlation was also observed between the age of the patients and the average colony count of *Candida albicans* in Groups 1 and 3. This finding implies that as individuals age, the colonization of *Candida albicans* also escalates, resulting in a higher likelihood of developing denture stomatitis and oral candidiasis. This association was particularly prominent in patients with severe COVID-19. A strong positive association was observed between the length of time dentures in all cohorts and the average number of *Candida albicans* colonies, as shown in Tables 4-6.

**Table 4:** Correlation between age, mean colony-forming units, and duration of denture use using Pearson’s correlation coefficient (r value) in Group 1 (mild and moderate COVID-19) *r value=0.00-0.199: Very weakly positive; **r value=0.20-0.39: Weakly positive; ***r value=0.8-1: Very strongly positive

Variables	Age	Mean colony forming units
Mean colony forming units	0.084*	-
Duration of denture use	0.204**	0.902***

**Table 5:** Correlation between age, mean colony forming units, and duration of denture use using Pearson’s correlation coefficient (r value) in Group 2 (severe COVID-19) *r value=0.40-0.599: Moderately positive; **r value=0.60-0.799: Strongly positive

Variables	Age	Mean colony forming units
Mean colony forming units	0.526*	-
Duration of denture use	0.486*	0.766***

**Table 6:** Correlation between age, mean colony-forming units, and duration of denture use using Pearson’s correlation coefficient (r value) in Group 3 (control group) *r value=0.00-0.199: Very weakly positive; **r value=0.20-0.39: Weakly positive; ***r value=0.8-1: Very strongly positive

Variables	Age	Mean colony forming units
Mean colony forming units	0.172*	-
Duration of denture use	0.274**	0.928***

## Discussion

Denture stomatitis is a prevalent issue that is often associated with denture use. The underlying causes of this problem can be attributed to the use of ill-fitting or poorly fitting complete denture prostheses, which can result in trauma to the underlying mucosa. Additionally, improper maintenance protocols followed by the wearer can contribute to the development of denture stomatitis [10]. *Candida albicans* is the most common causative agent of denture stomatitis.

Patients with COVID-19 display heightened susceptibility to contracting fungal infections, with the most prevalent being mucormycosis. This particular infection has been subjected to comprehensive examination in these individuals [11], whereas candidiasis, the second most common fungal infection, has received comparatively less research attention. Therefore, the present study was conducted to assess the presence of *Candida albicans* in COVID-19 denture wearers compared with healthy denture wearers. Various media have been used for speciation. Among these, agar plates containing Sabouraud dextrose have been demonstrated to be prompt, precise, and cost-effective. Specifically, this medium contains a chromogenic β-glucosaminidase substrate that generates dissimilarly pigmented compounds upon degradation by the species' corresponding enzymes, staining colonies of *Candida albicans* light green [6].

The present study revealed that maximum colonies of *Candida albicans* were found in patients with a history of severe COVID-19 infection, who were hospitalized for that, followed by patients who had mild to moderate COVID-19 infection and received at-home treatment, compared with the control group who did not have COVID-19 infection. This showed an association between COVID-19 caused by SARS-CoV-2 and *Candida albicans* in denture wearers. The existence of candidemia and candiduria within a COVID-19 unit located in Florida, United States, has instigated a comprehensive survey to determine the point prevalence. This survey revealed that 35 patients harbored *Candida albicans* [12].

The prevalence of Candida infection among geriatric individuals who use maxillary complete dentures may reach 65% [5]. This phenomenon can be attributed to the fact that the prosthesis enveloping the mucosa creates a microenvironment that is favorable for the proliferation of Candida, characterized by conditions such as a diminished pH level and an anaerobic setting. Hospitalized individuals exhibit an enhanced propensity for oral candidiasis because of environmental and systemic modifications that disrupt the indigenous microbiota and facilitate the development of opportunistic infections [7]. Currently, insufficient consideration has been given to the prevalence of opportunistic fungal infections in individuals afflicted with COVID-19 and the consequent complications that may arise.

Patients who have been afflicted by the severe acute respiratory syndrome coronavirus 2 (SARS-CoV-2) are at risk of developing a condition known as lymphocytopenia, which refers to a decrease in the number of lymphocytes in the blood. According to our study, *Candida albicans *was found to be more prevalent in severe COVID-19 patients which might be due to the fact that these patients were subjected to various medical interventions as part of their treatment, including hospitalization, administration of broad-spectrum antibiotics, corticosteroids, and antiviral drugs. It is crucial to highlight that these treatments, although necessary, can potentially further compromise the patients' immune systems and consequently increase their vulnerability to acquiring infections [13]. Souza et al. conducted a comprehensive investigation of the correlation between microorganisms present in the tracheal aspirate and oral biofilm of patients [14]. The findings revealed that a significant number of patients (59.37%) exhibited the same species of pathogens in both their tracheal aspirates and oral biofilms. This statistical evidence indicates a clear association between the presence of microorganisms in tracheal and mouth samples. Specifically, the study highlighted *Candida albicans* as a prominent microorganism that demonstrated this significant correlation.

When considering the treatment of severe COVID-19 patients, it is important to consider various systemic factors, including the administration of steroids, antiviral drugs, and broad-spectrum antibiotics. These factors, when combined with local factors such as wearing dentures and the reduction of salivary flow due to medication, as well as improper oral hygiene habits, can contribute to the growth and proliferation of *Candida albicans*. The interaction between these systemic and local factors creates an environment conducive to the growth and colonization of this fungal species [13]. In COVID-19 patients, the presence of invasive Candida infections results in a significant burden of comorbidity.

Infection with SARS-CoV-2 results in modifications of the immune and metabolic responses within individuals, thereby generating an inflammatory milieu that greatly facilitates the occurrence of fungal infections. Nevertheless, understanding the intricate mechanisms underlying this phenomenon is challenging. SARS-CoV-2 specifically targets cellular entities that express angiotensin-converting enzyme 2 (ACE2) and transmembrane protease serine 2 (TMPRSS2) [15]. Upon viral infiltration, damaged epithelial cells may also express apical receptors, such as integrins, which subsequently facilitate the interaction with proteins situated on the outermost layer of the Candida cell wall. These proteins, namely spore-coating (Cot H) proteins and mannoproteins, effectively endorse the adherence and invasion of fungal entities [16].

Hyperinflammation is a hallmark of COVID-19 and is characterized by high levels of circulating proinflammatory cytokines, including interleukin-6 (IL-6), interferon-γ (IFN-γ), IL-1β, tumor necrosis factor (TNF), acute phase reactants, and ferritin [15]. The activation of antiviral immunity after viral recognition by innate immune cells might paradoxically promote systemic inflammatory reactions and establish a highly permissive environment for the development of fungal co-infections by potentiating the expression of specific virulence factors and damage to the host. Owing to the imperative of combating hyperinflammation that is emblematic of severe COVID-19, the prevailing therapeutic interventions for severe COVID-19 lie in the realm of immunomodulatory pharmaceuticals, including corticosteroids and cytokine antagonists, such as tocilizumab. Despite their pertinence to the management of COVID-19, these medications impede the activation of both innate and adaptive antimicrobial responses and consequently serve as pivotal predisposing factors to secondary fungal infections [17].

Our investigation further revealed a positive correlation between age, duration of denture usage, and colonies of *Candida albicans*. The augmented presence of *Candida albicans* has been linked to advanced age, particularly over 60 years [18]. The prevalence of this fungal infection is also heightened by extended denture wear, including nocturnal usage and prolonged years of utilization [19,20]. This occurrence may be attributed to repetitive friction from the denture and escalated accumulation of plaque, thereby favoring the colonization of *Candida albicans* [20]. Gender disparities were not assessed in our research because of the similarity in age between males and females. This finding aligns with the observations made by Loster et al., who also reported no significant sex differences in a sample of comparable age [21].

Clinical implications of the study

Examination of the oral cavity is important in hospitalized patients diagnosed with COVID-19 because it plays a pivotal role in reducing morbidity and mortality rates caused by opportunistic infections, particularly denture candidiasis. In situations where the diagnosis reveals uncomplicated oral candidiasis, enhanced oral cleanliness and application of topical antifungal agents typically serve as appropriate therapeutic interventions.

Limitations of the study

The major constraints of the current investigation encompassed the case-control configuration, relatively limited sample size, and absence of assessment regarding gender disparities. Additional constraints of the investigation included the exclusion of COVID-19 patients who underwent intubation or mechanical ventilation. Moreover, the patients were evaluated solely 8-9 months after the COVID-19 infection. Further investigations incorporating more extensive study populations and longitudinal cohorts are indispensable to elucidate the correlation between *Candida albicans* and COVID-19 in individuals wearing dentures.

## Conclusions

Based on the findings of the current investigation, it can be posited that *Candida albicans*, the primary causative agent of candidiasis infection related to dentures, has the potential to exert a detrimental influence on individuals afflicted with COVID-19, thereby augmenting the morbidity and mortality associated with this viral infection. Medical personnel operating within hospital settings can derive substantial advantages from cultivating heightened awareness pertaining to the adverse consequences that suboptimal oral health can engender in patients affected by COVID-19. The outcomes delineated in this particular study underscore the criticality of incorporating oral healthcare providers within intensive care units to guarantee the provision of adequate treatment to aged patients with dental prostheses, as well as other individuals with compromised immune systems who have contracted COVID-19.

## References

[1] Singhal T (2020). A review of coronavirus disease-2019 (COVID-19). Indian J Pediatr.

[2] Atukorallaya DS, Ratnayake RK (2021). Oral mucosa, saliva, and COVID-19 infection in oral health care. Front Med (Lausanne).

[3] Baghizadeh Fini M (2020). Oral saliva and COVID-19. Oral Oncol.

[4] Peng X, Cheng L, You Y (2022). Oral microbiota in human systematic diseases. Int J Oral Sci.

[5] Shi B, Wu T, McLean J (2016). The denture-associated oral microbiome in health and stomatitis. mSphere.

[6] Manikandan S, Vinesh E, Selvi DT, Kannan RK, Jayakumar A, Dinakaran J (2022). Prevalence of candida among denture wearers and nondenture wearers. J Pharm Bioallied Sci.

[7] Dupont PF (1995). Candida albicans, the opportunist. A cellular and molecular perspective. J Am Podiatr Med Assoc.

[8] Ahmed N, Mahmood MS, Ullah MA, Araf Y, Rahaman TI, Moin AT, Hosen MJ (2022). COVID-19-associated candidiasis: possible patho-mechanism, predisposing factors, and prevention strategies. Curr Microbiol.

[9] Cuschieri S (2019). The STROBE guidelines. Saudi J Anaesth.

[10] Bachtiar BM, Fath T, Widowati R, Bachtiar EW (2020). Quantification and pathogenicity of Candida albicans in denture-wearing and nondenture-wearing elderly. Eur J Dent.

[11] Singh AK, Singh R, Joshi SR, Misra A (2021). Mucormycosis in COVID-19: A systematic review of cases reported worldwide and in India. Diabetes Metab Syndr.

[12] White PL, Dhillon R, Cordey A (2021). A national strategy to diagnose coronavirus disease 2019-associated invasive fungal disease in the intensive care unit. Clin Infect Dis.

[13] Jerônimo LS, Esteves Lima RP, Suzuki TY, Discacciati JA, Bhering CL (2022). Oral candidiasis and COVID-19 in users of removable dentures: is special oral care needed?. Gerontology.

[14] Souza LC, Mota VB, Carvalho AV, Corrêa RD, Libério SA, Lopes FF (2017). Association between pathogens from tracheal aspirate and oral biofilm of patients on mechanical ventilation. Braz Oral Res.

[15] Hoenigl M, Seidel D, Sprute R (2022). COVID-19-associated fungal infections. Nat Microbiol.

[16] Hadjadj J, Yatim N, Barnabei L (2020). Impaired type I interferon activity and inflammatory responses in severe COVID-19 patients. Science.

[17] Schwartz MD, Emerson SG, Punt J, Goff WD (2020). Decreased naïve T-cell production leading to cytokine storm as cause of increased COVID-19 severity with comorbidities. Aging Dis.

[18] Zaremba ML, Daniluk T, Rozkiewicz D (2006). Incidence rate of Candida species in the oral cavity of middle-aged and elderly subjects. Adv Med Sci.

[19] Prakash B, Shekar M, Maiti B, Karunasagar I, Padiyath S (2015). Prevalence of Candida spp. among healthy denture and nondenture wearers with respect to hygiene and age. J Indian Prosthodont Soc.

[20] Sumi Y, Kagami H, Ohtsuka Y, Kakinoki Y, Haruguchi Y, Miyamoto H (2003). High correlation between the bacterial species in denture plaque and pharyngeal microflora. Gerodontology.

[21] Loster JE, Wieczorek A, Loster BW (2016). Correlation between age and gender in Candida species infections of complete denture wearers: a retrospective analysis. Clin Interv Aging.

